# CT-Guided Aspiration of a Hemorrhagic Tarlov Cyst for the Treatment of a *Post-Partum* Sciatica: A Case Report and a Review of the Literature

**DOI:** 10.3389/fsurg.2022.788786

**Published:** 2022-07-12

**Authors:** Nicolas Serratrice, Sarkis Taifour, Christian Attieh, Joe Faddoul, Bilal Tarabay, Yassine Yachou, Moussa A. Chalah, Samar S. Ayache, Georges Naïm Abi Lahoud

**Affiliations:** ^1^ ICVNS - CMC Bizet, Paris, France; ^2^ CIMOP - CMC Bizet, Paris, France; ^3^ Centre Hospitalier de la Côte Basque, Bayonne, France; ^4^ Univ Paris Est Créteil, Excitabilité Nerveuse et Thérapeutique (ENT), Créteil, France; ^5^ AP-HP, Henri Mondor University Hospital, Department of Clinical Neurophysiology, DMU FIxIT, Créteil, France

**Keywords:** tarlov cyst, perineurial cyst, computed tomography guidance, puncture, aspiration, sciatica

## Abstract

**Background:**

Tarlov or perineural cysts are dilations of nerve roots resulting from a pathologically increased cerebrospinal fluid pressure. Although it is very common in the general population, most of these cysts remain asymptomatic. In some cases, they can evolve and exert pressure on neural elements, independently from their initial size.

**Case report:**

In this paper, we describe the case of a 33-year-old female known to have asymptomatic multiple and large radicular and pelvic Tarlov cysts. One cyst located in the right pelvic space progressed acutely after delivery, inducing a painful sciatica without neurological deficit. The intracystic bleeding can be a direct consequence of the delivery, leading to an acute and mechanical local compression of the right S1 root. A CT-guided puncture and aspiration allowed a complete recovery. This case report was completed by a review of the literature of these rare intracystic Tarlov bleedings.

**Conclusions:**

Intracystic hemorrhage is a rare complication of Tarlov cysts. Delivery-induced cyst bleeding was not described before. Patients known to have large and multiple Tarlov cysts should be monitored in post-partum, as their presence is considered a risk factor. Percutaneous cyst aspiration seems to be an effective and safe treatment to relieve symptoms.

## Introduction

Tarlov or perineural cysts are dilations of nerve roots resulting from pathologically increased cerebrospinal fluid (CSF) pressure (1). The cysts are most commonly observed in the sacral region, followed by thoracic, cervical, and lumbar regions (2, 3). In the majority of cases, a single cyst was found (2, 3). These cysts are present in 9% of the population, and are more common in women (2, 3). Most of these cysts remain asymptomatic throughout the patient's life, but some of them, independently of their size, can exert pressure on neural elements inducing pain, single or multiple radiculopathies of the *cauda equina*, and can be associated to fibromyalgia and chronic fatigue syndrome (1, 4). Tarlov cysts were also reported to be highly correlated with genitopelvic dysesthesia (specifically, “Persistent Genital Arousal Disorder”) (5), which was resolved by surgical treatment of the cysts (6). However, there are still no guidelines on the appropriate therapeutic management of Tarlov or perineural cysts: although the surgical interventions are associated with higher post-procedural complication rates, long-term efficacy, and success in terms of cyst resolution seem to be superior following surgery compared to percutaneous procedures in the management of symptomatic cysts (7, 8).

## Case Report

**Figure 1:**
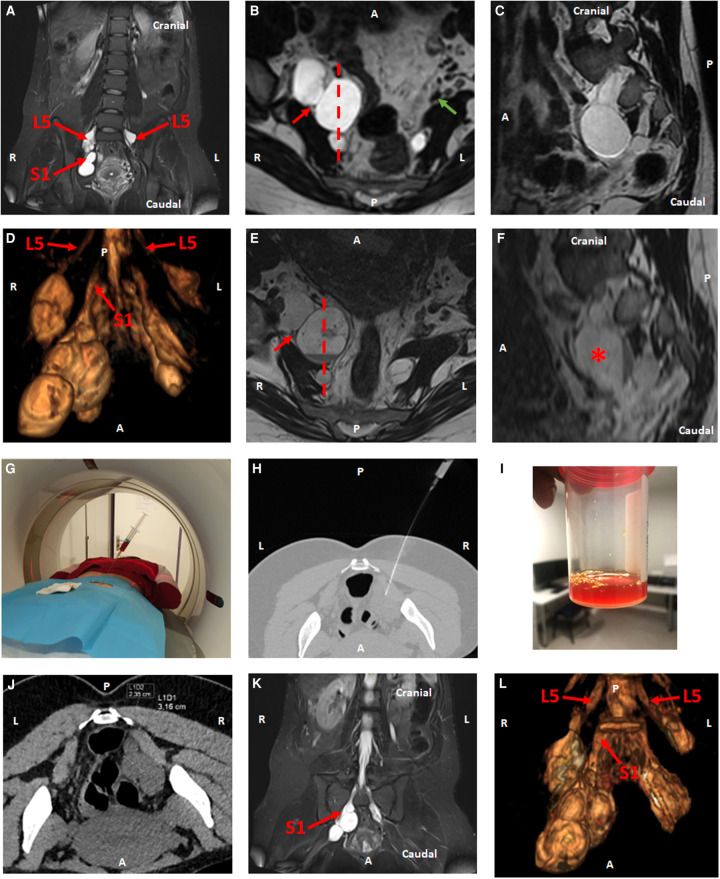
Presence of large bilateral sacral radicular cysts on MRI coronal (**A**), axial (**B**) and sagittal (**C**) T2-weighed sequences predominantly on the right L5 and S1, S2 and S3 roots (red arrows) and on the left L5 root (**A**). (**C**) correspond to dotted plane in (**B**). (**B**) Pelvic cystic formation of 36 × 29 mm on the right S1 nerve root (MRI axial view T2-weighed sequence), right (red arrow) and left (green arrow) sciatic nerves. (**D**) 3D reconstruction of the different cysts associated with the different nerve roots. Fluid-fluid level, on axial (**E**) and sagittal (**F**) T2-weighed sequences. (**F**) correspond to dotted plane in (**E**). Bleeding was noticed predominantly on the right L5 and S1 cysts (red asterisk) and appearing to sheath the distal portion of right S1 (red arrow). This intracystic hemorrhagic complication was probably due *post-partum* (**G,H**) Aspiration of a hemorrhagic liquid under pelvic-abdominal CT-scan guidance. (**I**) Fluid aspect. (**J**) Dimensions of this hemorrhagic cyst immediately after aspiration (CT-scan axial view): 31 × 25 mm. (**K**) MRI control 1 year after the procedure, with 3D reconstructions (**L**). Remodeling of the cyst was observed. A reduction of 7% of its initial volume was determined by volumetric analysis before and after the procedure, and the S1 root is better visualized. R = Right, L = Left, A = Anterior, P = Posterior.

A 33-year-old female known to have asymptomatic multiple and large radicular and pelvic Tarlov cysts extending from L5 to S3 roots discovered in 2016 on an MRI done for low back pain (Figures 1A, B), was admitted to our center 7 days after delivery for painful S1 right sciatica (intensity of 10 on visual analogic scale (VAS)). The patient presented without neurological motor deficit, especially on ankle plantar flexors and hip extensors testing. Achilles reflex was still present. In a supine position, no positive straight leg raise or crossed straight leg raise was observed, and in a seated position, Bechterew or slump tests were both negative. She has also no genitopelvic sensory and/or motor dysfunction (clitoral, vaginal, urinary bladder, or bowel discomfort and/or dysfunction), corresponding to pudendal and pelvic nerve testing. Magnetic resonance imaging (MRI) showed a large pelvic cystic formation of 36 × 29 mm on the right S1 nerve root (Figures 1D–F), with a fluid-fluid level corresponding to a *post-partum* hemorrhagic complication and compressing the distal portion of right S1 nerve. No visible focal intramedullary signal abnormality was observed. The *Conus terminalis* was located at the level of the L1 vertebrae. A low-grade lumbar osteoarthritis was noted between L3 and S1, predominantly in L4-L5 without notable root. The intracystic hemorrhage was responsible for a global increase in the volume of the cyst compared to the previous MRI, explaining the sciatica. After a multidisciplinary discussion, a CT-guided puncture-aspiration of the cyst under local anesthesia was decided (Figures 1G, H). The procedure was performed without any complications; 8 cc of a hemorrhagic fluid was aspirated (Figure 1I), relieving the patient's sciatica immediately (intensity of zero on the VAS scale after the procedure). No sealing procedure was performed to limit the risk of intracystic rebleeding, and to avoid the risk of recompression due to the cyst filling on the S1 root. (Figure 1J) illustrates the decompression of the sciatic verve concomitant with the cyst shrinkage after aspiration (31 × 25 mm). The effects on pain were immediate. No neurological deterioration occurred after the procedure, and specifically on the genitopelvic sensory and/or motor function. The patient was still pain-free (VAS zero) at 12 months follow-up, and the Tarlov cyst did not fill-up on the MRI follow-up (Figures 1J, K). Remodeling was observed at the level of the aspirated cyst. A volume analysis pre/post-procedure showed that the cyst had lost 7% of its initial volume, and the S1 root is better visualized.

## Discussion

Here, we described the case of a 33-year-old female patient known with asymptomatic large pelvic Tarlov cysts impinging on nerve roots including S1–3, that became symptomatic after a *post-partum* intracystic hemorrhage within the largest cyst. A CT- guided puncture eliminated the sciatica and allowed long-term resolution of symptoms. To our knowledge, this is the first time that this management option is described in the literature.

Symptomatic Tarlov cysts are not frequent and cases of intracystic hemorrhage appear to be extremely rare (2, 3, 9). Hemorrhagic cysts are mainly described as complications of trauma or anticoagulation therapies (10). To the best of our knowledge, this is the first reported case of delivery-induced bleeding within a previously asymptomatic Tarlov cyst leading to sciatica. In our patient, delivery was the traumatic pelvic event. In case of rupture, they can be responsible of intraspinal hemorrhage (11). Subarachnoid hemorrhage (SAH) resulting from bleeding into subarachnoid space can also be the source of the development of pain in previously asymptomatic Tarlov cysts, by local irritation of the nerve roots (12). In extremely rare cases, an intracystic bleeding can be found (12).

We hypothesized that the intracystic post-delivery bleeding was responsible for the increase in its volume compared to the previous MRI done in 2016, and therefore for the compression on the right S1 root. This is the reason why we decided to perform a percutaneous therapeutic procedure rather than an open surgical intervention. The supracentimetric size of the cyst was an additional factor to this choice. To increase the precision and avoid breaking the nearby cysts, we performed the puncture of the hemorrhagic cyst directly under CT-scan guidance. Regarding the nearby cysts, we decided not to puncture them to avoid any rupture, and therefore avoid any risk of additional complications. The percutaneous treatment provided immediate and lasting relief. In this case, the intracystic bleeding was a direct consequence of the delivery, inducing an acute mechanical local compressive complication. We advise, when possible, to use targeted percutaneous techniques in this kind of clinical presentation, with potential excellent functional outcome.

It is common for Tarlov cysts to fill up with CSF again after aspiration. In this case, remodeling was observed, and the cyst did not fill-up. We decided not to include any form of sealing of the cyst after aspiration to avoid the rebreeding risk. Although glue injection has been described in previous reports to prevent re-filling, we opted not to use it in our case to prevent the reconstitution of the compressive volume of the cyst on nerve S1 nerve root with filling agents (13–15).

## Conclusions

We described the case of a 33-year-old female known to have asymptomatic multiple and large pelvic Tarlov cyst. A delivery-induced intracystic bleeding led to an acute and mechanical local compression of the right S1 root, causing a painful sciatica without neurological deficit. A direct CT-guided puncture and aspiration of the cyst allowed a complete recovery and long lasting efficiency. Hence, percutaneous techniques may be the treatment of choice in this situation, with potential good functional outcome. Female patients known to have large multiple Tarlov cysts should be monitored in post-partum to manage adequately this rare complication.

## Data Availability

The original contributions presented in the study are included in the article/Suplementary Material, further inquiries can be directed to the corresponding author/s.
